# Supplementary material for: Convergent evolution of distinct D-ribulose utilisation pathways in attaching and effacing pathogens
Source: Nat Commun. 2025 Jul 29;16:6976. doi: 10.1038/s41467-025-62476-5 (PMC12307932; doi:10.1038/s41467-025-62476-5)
Supplement: Supplementary file 1 — Supplementary Information [file 41467_2025_62476_MOESM1_ESM.pdf]

Supplementary information

Convergent evolution of distinct D-ribulose utilisation pathways in attaching and effacing pathogens

Curtis Cottam, Kieran Bowran, Rhys T. White, Arnaud Baslé, Inokentijs Josts and James PR Connolly

Supplementary Figures 1-12

Supplementary table 1 – Bacterial strains used in this study.

Supplementary table 2 – Primers used in this study.

Supplementary table 3 – Plasmids used in this study.

Supplementary table 4 – X-ray data collection and refinement statistics

Supplementary references

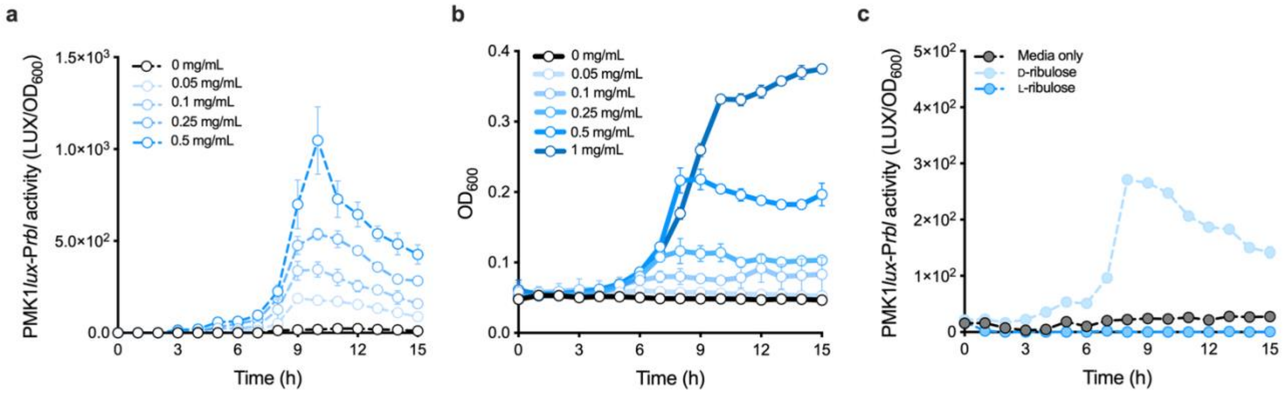

Supplementary Fig. 1. The *C. rodentium ROD_24811-61* locus is responsive to a range of D-ribulose concentrations. **a,** Transcriptional reporter assay of *C. rodentium* transformed with the pMK1*lux*-P_{24811} plasmid cultured in MEM-HEPES alone or supplemented with the indicated concentration range D-ribulose. **b,** Growth analysis of wild type *C. rodentium* in M9 minimal media supplemented with the indicated concentration range of D-ribulose as a sole carbon source. Error bars for reporter assays and growth curves represent the standard deviation of the mean from three independent experiments (*n* = 3 biological replicates). **c,** Transcriptional reporter assay of *C. rodentium* transformed with the pMK1*lux*-P_{24811} plasmid cultured in MEM-HEPES alone or supplemented with 0.1 mg/ml D-ribulose or L-ribulose

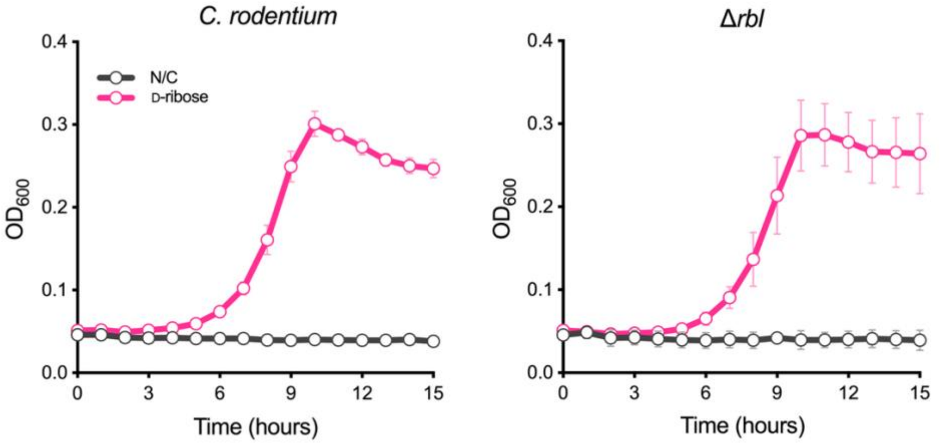

Supplementary Fig. 2. The *C. rodentium ROD_24811-61* locus is not required for growth on D-ribose. Growth analysis of wild type *C. rodentium* and Δ*rbl* cultured in M9 minimal media supplemented with 0.5 mg/mL D-ribose. The no sugar control indicates wild type *C. rodentium* inoculated into M9 without a carbon source. Error bars represent the standard deviation of the mean from three independent experiments (*n* = 3 biological replicates).

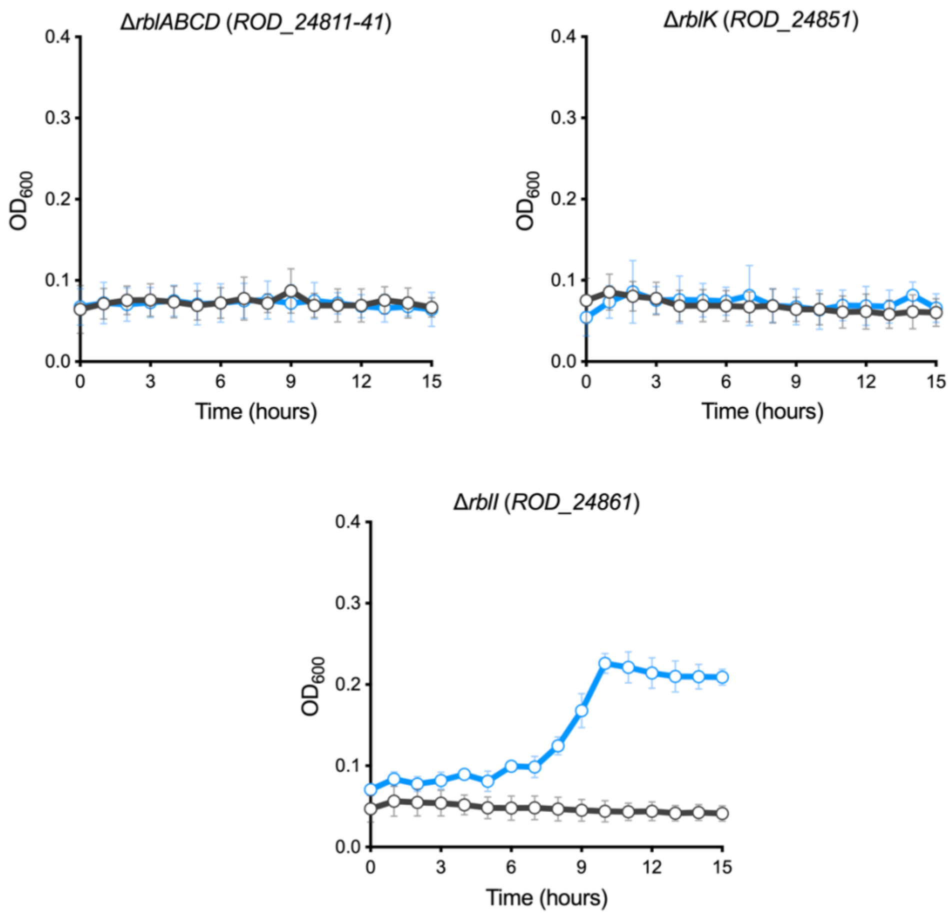

Supplementary Fig. 3. The Rbl ABC transporter and D-ribulokinase are required for utilisation of D-ribulose by *C. rodentium*. Growth analysis of wild type *C. rodentium,* Δ*rblABCD,* Δ*rblK* and Δ*rblI* cultured in M9 minimal media supplemented with 0.5 mg/mL D-ribose. The no sugar control indicates wild type *C. rodentium* inoculated into M9 without a carbon source. Error bars represent the standard deviation of the mean from three independent experiments (*n* = 3 biological replicates).

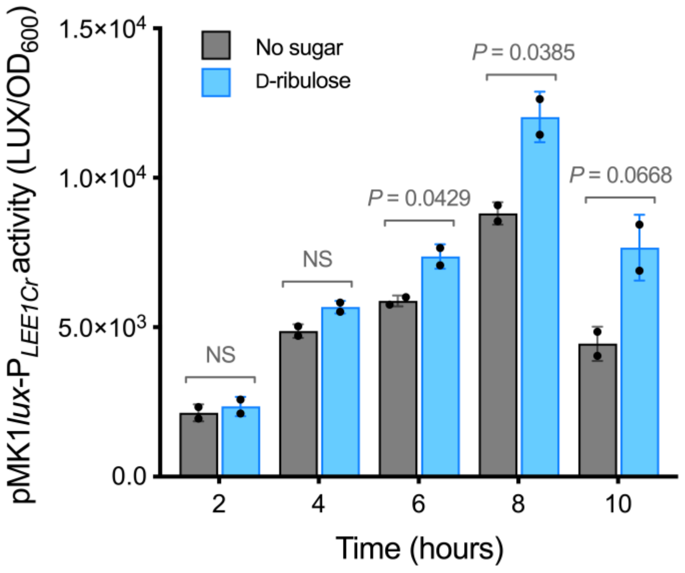

Supplementary Fig. 4. D-ribulose metabolism enhances expression of the LEE-encoded type 3 secretion system in *C. rodentium*. Transcriptional reporter analysis of *C. rodentium* transformed with pMK1*lux*-P$_{LEE1Cr}$ cultured in MEM-HEPES (grey) or supplemented with 0.5 mg/mL of D-ribulose (blue). Data are depicted as luminescence units (LUX) divided by optical density (OD_{600}) at each timepoint. Statistical significance was determined by two-tailed students' *t*-test. Error bars represent standard deviation of the mean (*n* = 2 biological replicates).

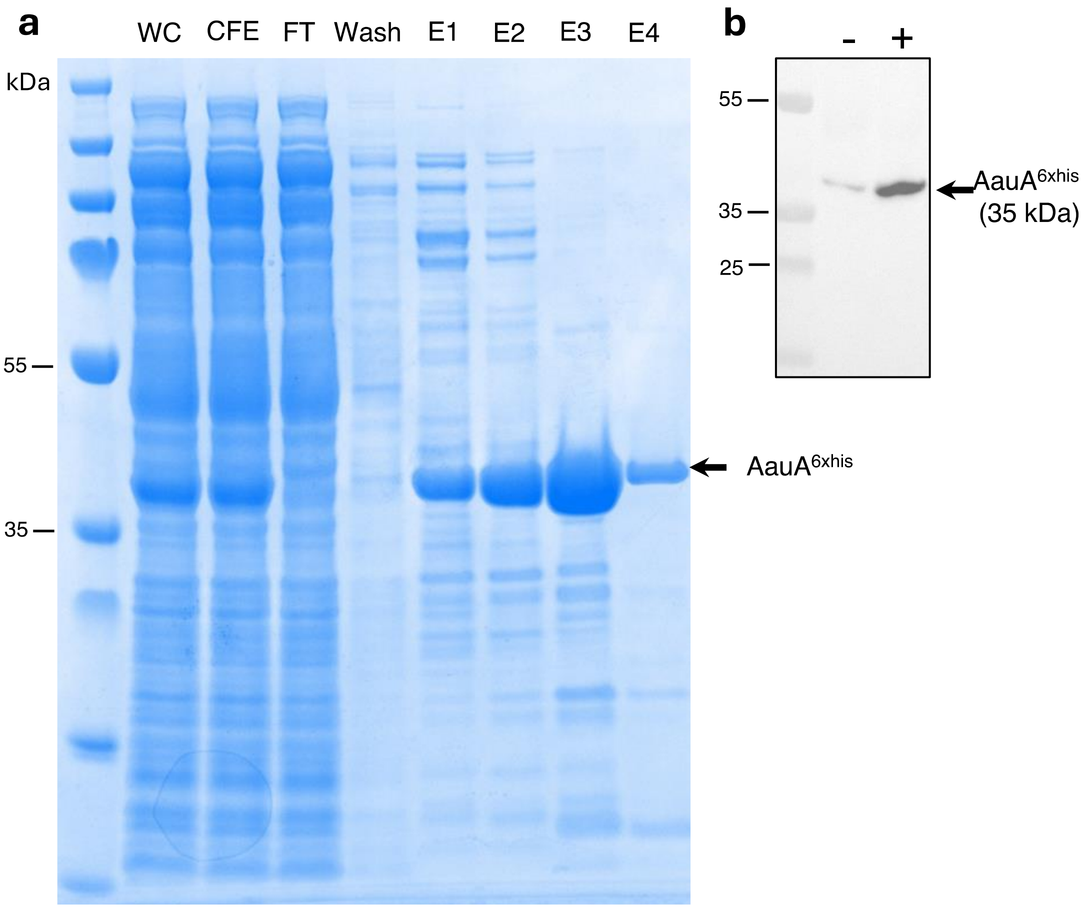

Supplementary Fig. 5. Overexpression and purification of recombinant AauA from EHEC. a, SDS-PAGE analysis of AauA with C-terminal His-tag purification by immobilized metal affinity chromatography. WC: Whole cell fraction; CFE: Cell free extract; FT: Flow through. E1 and E2 correspond to elution with 10 mM imidazole. E3 and E4 correspond to elution with 100 mM imidazole. **b,** Western blot detection of his-tagged AauA before and after induction with 0.5 mM IPTG. Purifications were performed and validated in two separate occasions.

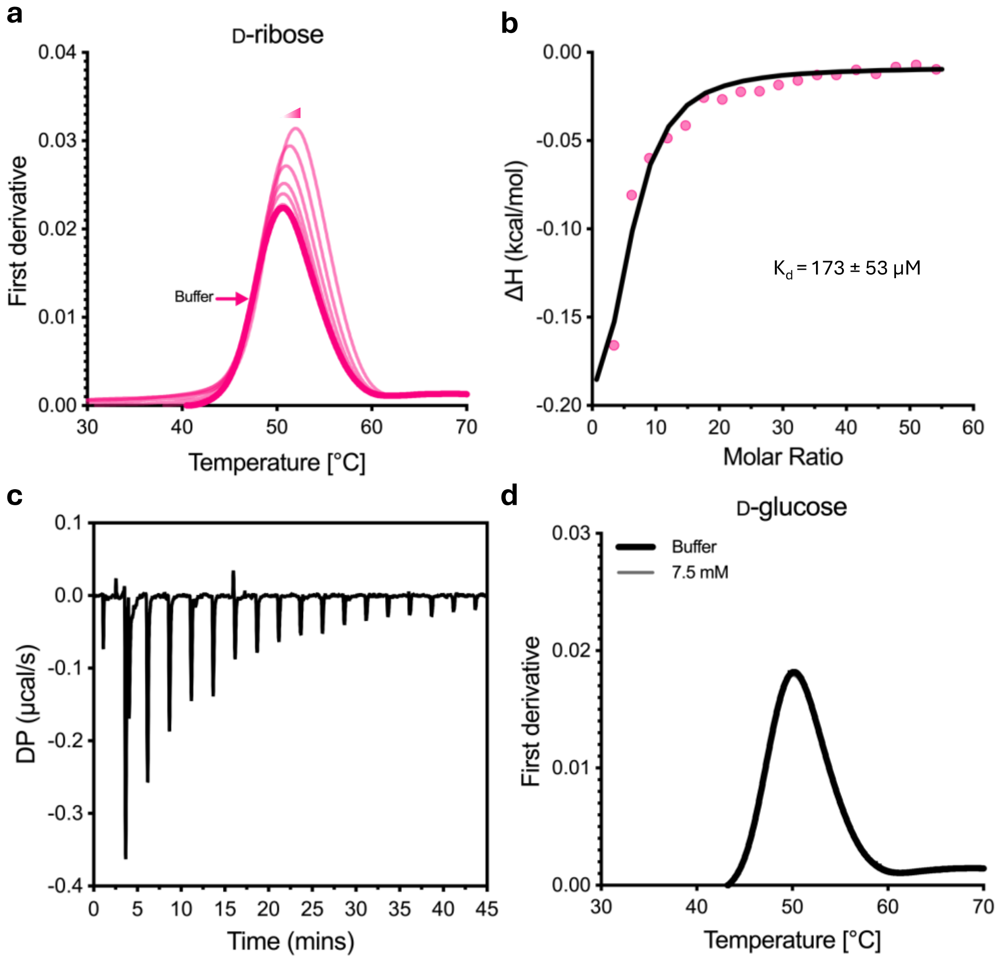

Supplementary Fig. 6. AauA shows low affinity for D-ribose. a, NanoDSF data depicting the shift in melting temperature (ΔT_m) of purified AauA in the presence of increasing concentrations of D-ribose (pink). The buffer only control is illustrated in bold. **b,** Representative ITC thermogram of D-ribose titrated into purified AauA, with the corresponding calculated K_d shown. **c,** The associated integration of heats derivative curve. **d,** NanoDSF control illustrating no shift in melting temperature (ΔT_m) of purified AauA in the presence of D-glucose (grey) over the buffer only control (black). NanoDSF and ITC experiments were performed in triplicate (*n* = 3).

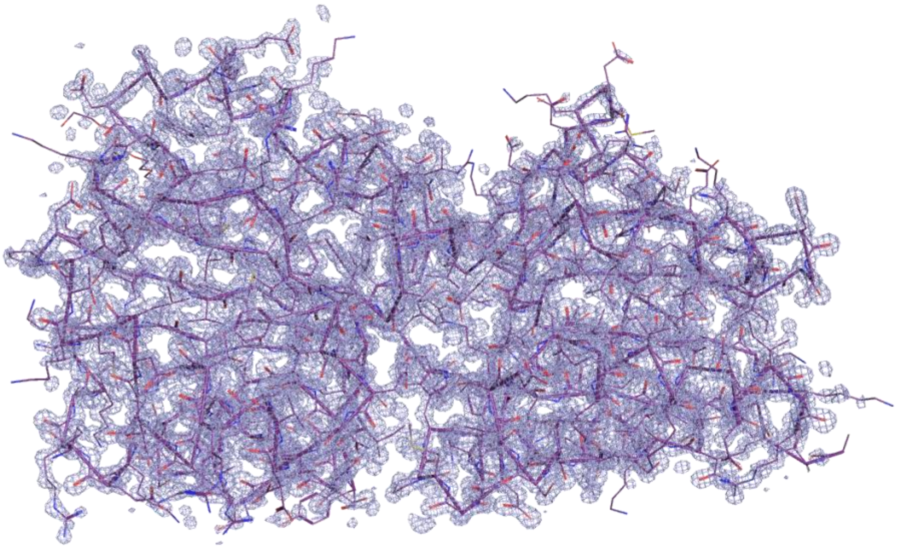

Supplementary Fig. 7. Electron density map of AauA bound D-ribulose. 2Fo-Fc map of AauA contoured at 2 sigma. Data corresponds to the structure presented in Fig. 2d.

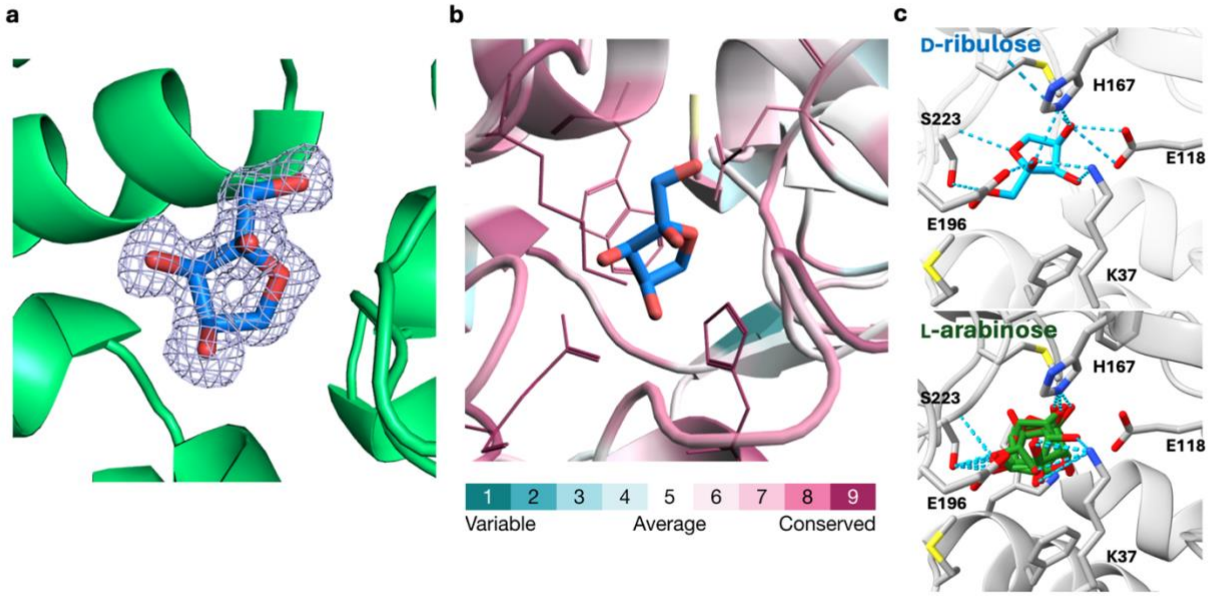

Supplementary Fig. 8. Conserved binding site of AauA coordinates D-ribulose with high specificity. a, Polder omit map (contoured at 3.5 σ) showing the conformation of alpha-D-ribulose within the binding pocket of AauA. **b,** Highly conserved nature of all coordinating residues within the substrate-binding site as analysed using ConSurf. **c,** Molecular docking of L-arabinose into the binding site of AauA (bottom panel) suggests the potential basis for the weaker affinity of L-arabinose over D-ribulose (top panel). Within the crystal structure, D-ribulose is well-coordinated within the binding site (top panel), while L-arabinose is potentially less well-coordinated. 6 different conformers of L-arabinose (obtained from PubChem) were docked into the binding pocket using AutoDock Vina into the crystal structure of AauA. D-ribulose was removed prior to docking.

```
24851      --MASYFIGVDVGTGSARAGVFDLNGRMV------GQATRAIEIYRPQADFVE------Q    46
3QDK       MSLTKYTIGVDYGTESGRAVLIDLSNGQELADHVTPYRHGVIDQYLPNTNIKLGHEWALQ    60
             ::.* **** ** *.** ::**..              .*: * *::::        *

24851      SSDNIWQAVCNAVRDAINQSDINPIQVKGLGFDA-TCSLVVLDKEGKPLTVSPSGRS--E    103
3QDK       HPLDYVEVLTTSVPAVMKESGVDADDVIGIGVDFTACTMLPVDEEGQPLCLLAQYKDNPH    120
              :  :.: .:*  .:::*.::  :* *:*.*  :*::: :*:**:** :  . :.  .

24851      QNIIVWMDHRAITQAERINALHH----RVLDYVGGIISPEMQTPKLLWLKQHMPNTWANA    159
3QDK       SWVKLWKHHAAQDKANAINEMAEKRGEAFLPRYGGKISSEWMIAKVWQILDEAEDVYNRT    180
           . : :* .* *  :*: ** : .     .*   ** ** *    *:  : :.  :.: .:

24851      GYYFDLPDFLTWRATGDDTRSLCSTVCKWTYMGHEDKWDASYFREIGLEDLLEH-DAAKI    218
3QDK       DQFLEATDWIVSQMTGKIVKNSCTAGYKAIWHKREGYPSNEFFKALD--PRLEHLTTTKL    238
           . :::  *::. : **. .:. *::  *  :  :*.  . .:*: :.    ***  ::*:

24851      GRYVKTMGEPLGHGLSQRAASEMGLIPGTAVSVSIIDAHAGTLGTLGASGVSGEVADFDR    278
3QDK       RGDIVPLGERA-GGLLPEMAEKMGLNPGIAVAVGNVDAHAAVPAV----GV-----TTPG    288
              :  :**    **  . *.:*** ** **:*. :****.. ..    **

24851      RIALIGGTSTGHMAISKEPRFIGGVWGPYYSAILPEYWLNEGGQSATGALIDHIIQSHPC    338
3QDK       KLVMAMGTSICHMLLGEKEQEVEGMCGVVEDGIIPGYLGYEAGQSAVGDIFAWFVKHGVS    348
           ::.:  ***  ** :.:: : : *: *   ..*:* *   *.****.* ::  :::   .

24851      YQTLLAQAKSQGQTIYEVLNALLRKMAGEPENIAFLTRDIHILPYFHGNRSPRANPTLTG    398
3QDK       AA-TFDEAQEKGVNVHALLEEKA-------SQLRPGESGLLALDWWNGNRSILVDTELSG    400
               : :*:.:* .:: :*:          .::     .:  * :::****  .:  *:*

24851      AISGLKLSRTPEDMALQYLATIQAIALGTRHIIETMNQSGYSIDTIMASGGGTK-NPIFV    457
3QDK       MLLGYTLQTKPEEI---YRALLEATAFGTRAIVDAFHGRGVEVHELYACGGLPQKNHLLM    457
            : * .*. .**::   * * ::* *:*** *::::   * .:. : *.**  : * :::

24851      QEHANATGCAMLLPEESEAMLLGGAMMGTIAAG----VFDTFPEAMSAMSRIGKTVTPQT    513
3QDK       QIFADVTNREIKVAASKQTPALGAAMFASVAAGSEVGGYDSIEEAAKKMGRVKDETFKPI    517
           * .*:.*.  : :  ..::  **.**:.::***     :*:: ** . *.*: . .

24851      NRIKHYYDRKYQVF---HEMYQDH----MKYRQLMQEA------L---------- 545
3QDK       PEHVAIYEKLYQEYVTLHDYFGRGANDVMKRLKALKSIQHRPSSLLTEGHHHHHH 572
            .    *:: ** :   *: :       **  : ::.       *
```

Supplementary Fig. 9. Sequence alignment of ROD_24851 (RblK) from *C. rodentium* with AraB (3QDK). Amino acid sequence alignment indicating the position of residues in the AraB substrate binding cleft that are known to interact with L-ribulose (blue). The red arrow indicates the only non-conserved binding residue (as illustrated in Fig. 5b).

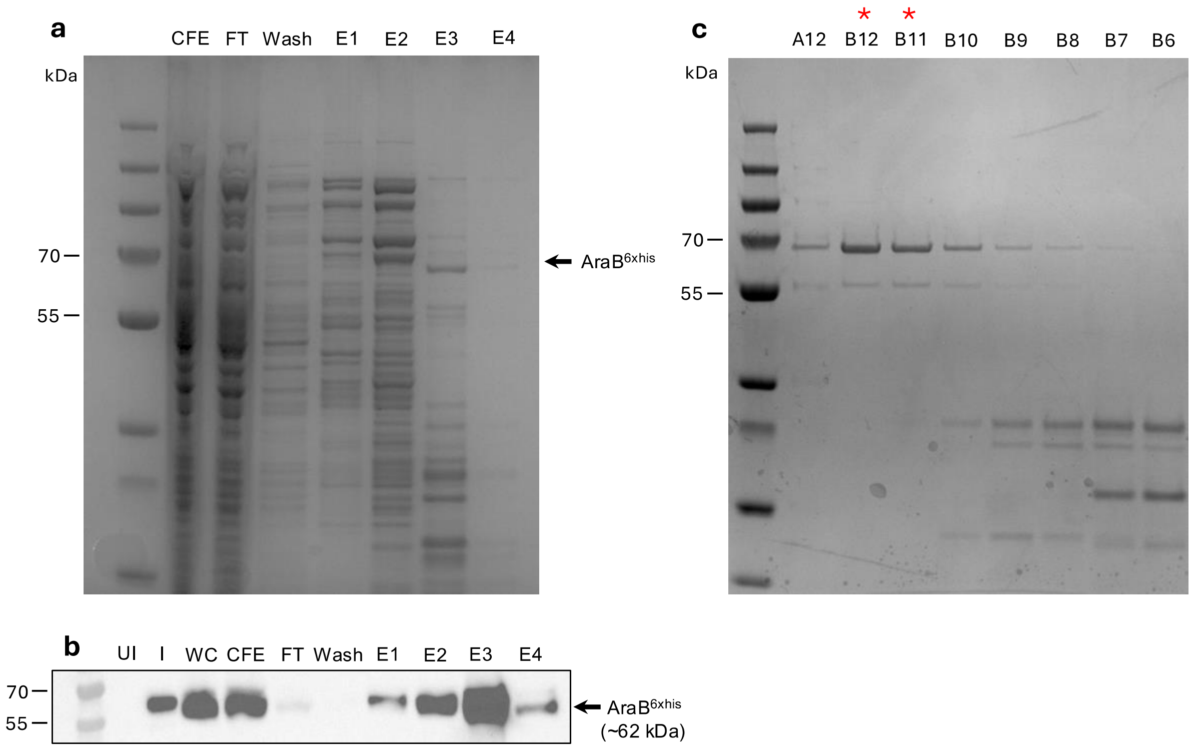

Supplementary Fig. 10. Overexpression and purification of recombinant AraB from EHEC. a, SDS-PAGE analysis of AraB with C-terminal His-tag ($AraB^{6xHis}$) purification by immobilized metal affinity chromatography. WC: Whole cell fraction; CFE: Cell free extract; FT: Flow through. E1 and E2 correspond to elution with 10 mM imidazole. E3 and E4 correspond to elution with 100 mM imidazole. **b,** Western blot detection of his-tagged AraB from the same fractions in panel **a**. Uninduced (UI) and induced (I) fractions are also shown. **c,** SDS-PAGE analysis of $AraB^{6xHis}$ further purified by size exclusion chromatography. The fractions indicated by the red asterisks were pooled and used for subsequent assays. Purifications were performed and validated in two separate occasions.

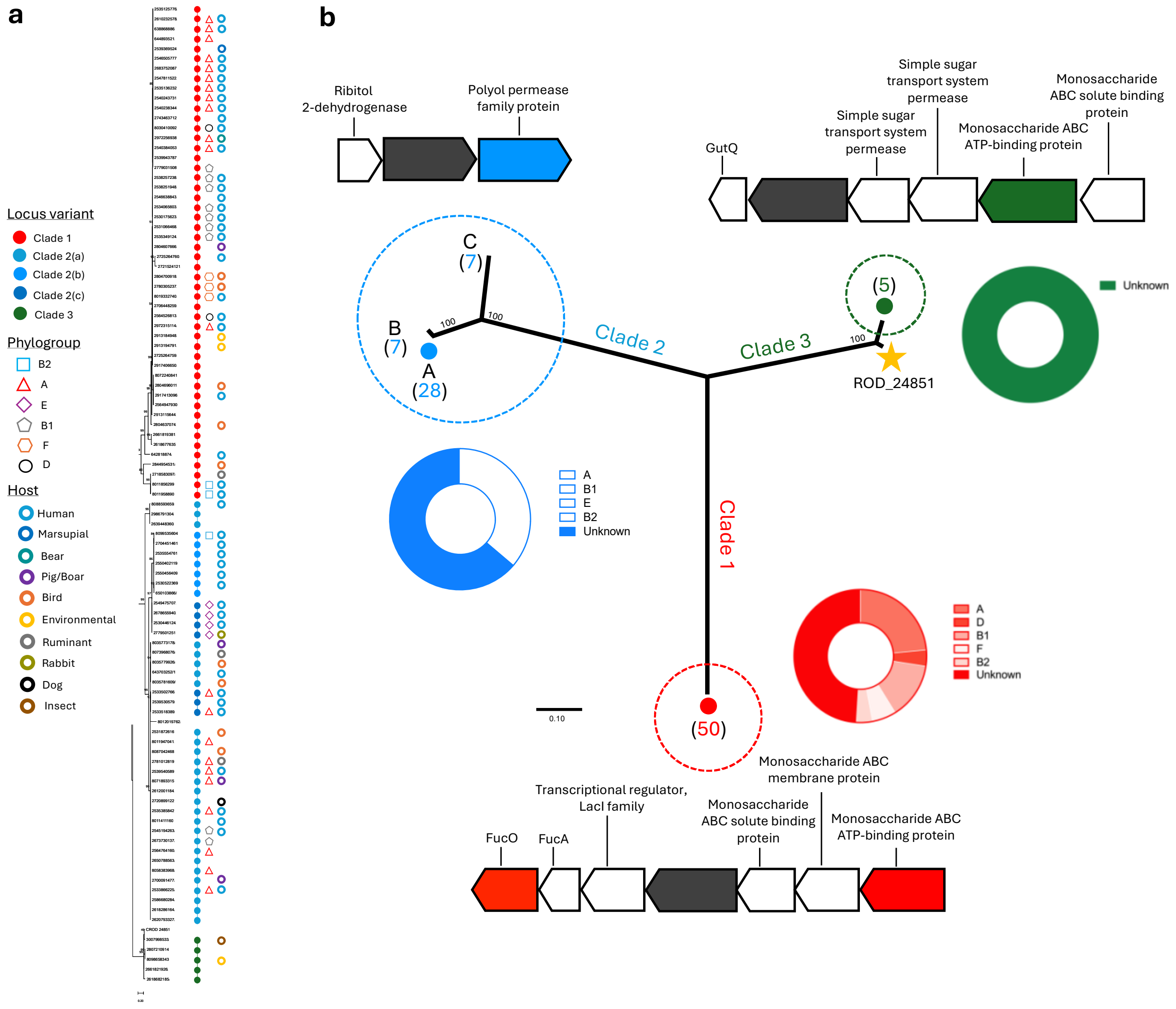

Supplementary Fig. 11. Analysis of D-ribulokinase carriage in *Escherichia* spp. a, Maximum likelihood phylogenetic tree of identified FGGY-family carbohydrate kinase (TIGR01315) homologues across *E. coli*, representative of 100 bootstrap replicates. Associated strain metadata from the Integrated Microbial Genomes and Microbiomes and Enterobase databases is displayed. **b,** Maximum likelihood phylogeny highlighting the three clades identified amongst strains carrying a predicted D-ribulokinase. Representative strains from each clade were used to generate the phylogeny. The organisation of loci associated with D-ribulokinase is shown, and the phylogroups strains were found to belong are summarised across each clade.

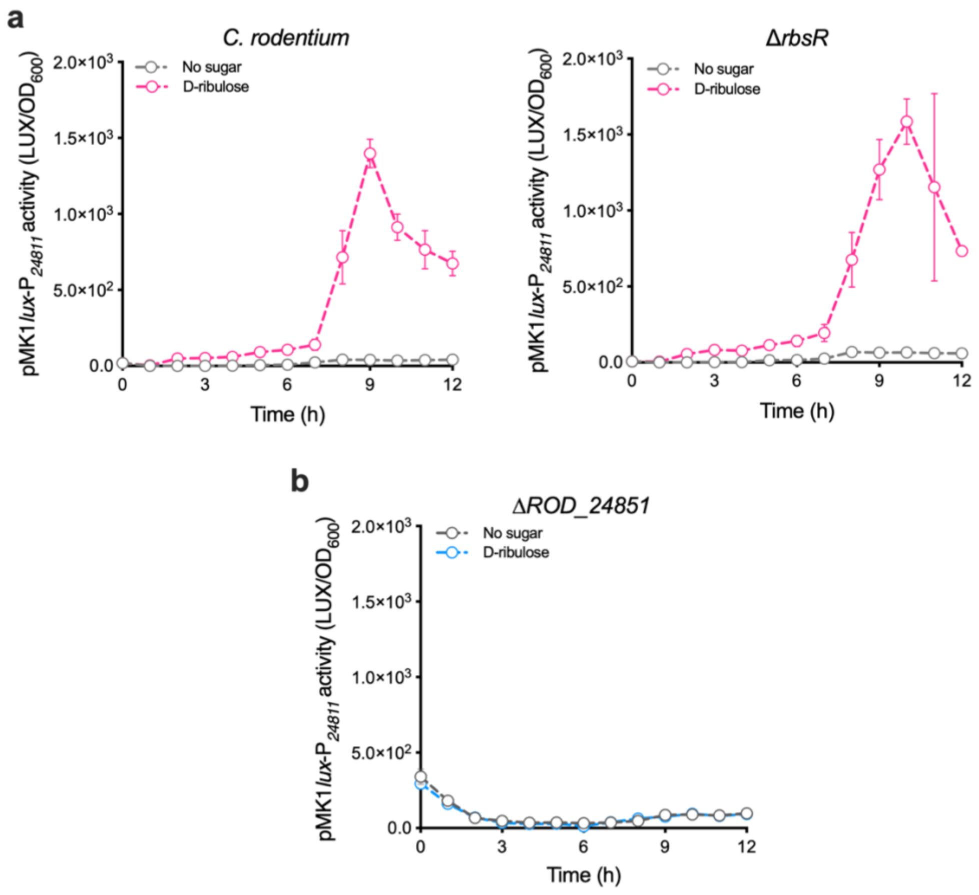

Supplementary Fig. 12. D-ribulose metabolism is required to activate transcription of *rbl*. a, Transcriptional reporter assay of wild type *C. rodentium* (left) and Δ*rbsR* (right) transformed with the pMK1*lux*-P_{24811} plasmid cultured in MEM-HEPES alone (grey) or supplemented with 0.5 mg/mL D-ribulose. **b,** pMK1*lux*-P_{24811} reporter assay in the *C. rodentium* Δ*rblK* mutant background, cultured in MEM-HEPES alone (grey) or supplemented with 0.5 mg/mL D-ribulose. Error bars for reporter assays represent the standard deviation of the mean from three independent experiments (*n* = 3 biological replicates).

Supplementary Table 1 – Bacterial strains used in this study.

**Strain**	**Description**	**Reference**
ZAP193	EHEC O157:H7 str. ZAP193 (NCTC 12900) Stx^{-}	Roe *et al*. 2004
Δ*aau*	ZAP193 *Z0415-9* deletion mutant; Kan^{R} $Strep^{R}$	Cottam *et al*. 2024
TUV93-0	EHEC O157:H7 str. EDL933 Stx^{-}	Campellone *et al*. 2002
Δ*araBAD*	TUV93-0 *araBAD* deletion mutant; Kan^{R}	Cottam *et al*. 2024
ICC169	*C. rodentium* O152 serotype; Nal^{R}	Petty *et al*. 2010
Δ*rbl*	ICC169 *rblABCDKI* (*ROD_24811-61*) deletion mutant; Kan^{R}	This study
Δ*rblABCD*	ICC169 *ROD_24811-41* deletion mutant; Kan^{R}	This study
Δ*rblK*	ICC169 *ROD_24851* deletion mutant; Cm^{R}	This study
Δ*rblI*	ICC169 *ROD_24861* deletion mutant; Kan^{R}	This study
Δ*rbsR*	ICC169 *rbsR* deletion mutant; Kan^{R}	This study

Supplementary Table 2 – Primers used in this study.

Primer Name	Description	Sequence
*ROD24811-51*_LRed_Fwd	ROD24811-51 KO forward primer	ttgctaacctcgtttcgtgacatgccctggtcccattaa aaggaacgacagtgtaggctggagctgcttc
*ROD24811-51*_LRed_Rev	ROD24811-51 KO reverse primer	cgcttcgctatagagccgccaggtggcggtggcctgct gccatgcgctgccatatgaatatcctccttag
*ROD24811-51*_Check_Fwd	ROD24811-51 KO check forward primer	tgccctggtcccattaaa
*ROD24811-51*_Check_Rev	ROD24811-51 KO check reverse primer	attaacgcctgccactgc
*ROD24811-41*_LRed_Fwd	ROD24811-41 KO forward primer	ttgctaacctcgtttcgtgacatgccctggtcccattaa aaggaacgacagtgtaggctggagctgcttc
*ROD24811-41*_LRed_Rev	ROD24811-41 KO reverse primer	ttcctacatcgacaccaataaaataactcgccatcat ttttctcccgaaacatatgaatatcctccttag
*ROD24811-41*_Check_Fwd	ROD24811-41 KO check forward primer	acatgccctggtcccattaa
*ROD24811-41*_Check_Rev	ROD24811-41 KO check reverse primer	ggtaaatttcaatggcgcgc
*ROD24851*_LRed_Fwd	ROD24851 KO forward primer	cctctttatcgcattacagaatcgtaaagcctgatttcg ggagaaaaatggtgtaggctggagctgcttc
*ROD24851*_LRed_Rev	ROD24851 KO reverse primer	cgcttcgctatagagccgccaggtggcggtggcctgct gccatgcgctgccatatgaatatcctccttag
*ROD24851*_Check_Fwd	ROD24851 KO check forward primer	atacggcgagtcctatctgc
*ROD24851*_Check_Rev	ROD24851 KO check reverse primer	ttatcatcaggtcgtcggca
*ROD24861*_LRed_Fwd	ROD24861 KO forward primer	gagatgtatcaggatcacatgaagtaccgtcagctga tgcaggaggcgttgtgtaggctggagctgcttc
*ROD24861*_LRed_Rev	ROD24861 KO reverse primer	tattttctgcatatcggaaaaagccccgtctatgggac ggggccaggccacatatgaatatcctccttag
*ROD24861*_Check_Fwd	ROD24861 KO check forward primer	cgcagaccaaccgcattaag
*ROD24861*_Check_Rev	ROD24861 KO check reverse primer	taacgtcaggattgcagggg
pMK1*lux*-PROD24811_Fwd	Forward primer for cloning ROD24811 promoter with EcoRI	cccgaattcctgccgcgactgctggca
pMK1*lux*-PROD24811_Rev	Reverse primer for cloning ROD24811 promoter with BamHI	cccggatccattgtcgttccttttaat
pMK1*lux*_Check_Fwd	Forward primer to check pMK1*lux* cloning	ctataaaaataggcgtatcac
pMK1*lux*_Check_Rev	Reverse primer to check pMK1*lux* cloning	ctggccgttaataatgaatg
pSU-PROM-*rbl*_Fwd	Rbl Gibson assembly forward primer	tctaccacagaggaggatccatgaaattcaaac tcgcattactac
pSU-PROM-*rbl*_Rev	Rbl Gibson assembly reverse primer	ctcgaggggtcgactctagatcataacgcctcct gcatc
pSU-PROM_Check_Fwd	Forward primer to check pSU-PROM cloning	ctcttcgctattacgccagc
pSU-PROM_Check_Rev	Reverse primer to check pSU-PROM cloning	accctcatcagtgccaacat
pSU-PROM_Linear_Fwd	pSU-PROM linearisation forward primer	tctagactcgacccctcg

pSU-PROM_Linear_Rev	pSU-PROM linearisation reverse primer	ggatcctcctctgtggtag
pET28a-araB_Fwd	Forward primer for cloning *araB* with X	ccatggatggcgattgcaattggcctcgattttggc
pET28a-araB_Rev	Reverse primer for cloning *araB* with X	ctcgagtagagtcggaacggcctgggcagcctgtgc
pET28a-aauA_Fwd	Forward primer for cloning *aauA* with X	ccatggatgatgaataaacgttttgttatc
pET28a-aauA_Rev	Reverse primer for cloning *aauA* with X	ctcgagataaagtgagtcgatattgtcttt
pET28a_Check_Fwd	Forward primer to check pET28a cloning	acccctcaagacccgtttag
pET28a_Check_Rev	Reverse primer to check pET28a cloning	atcggtgatgtcggcgatat

Supplementary Table 3 – Plasmids used in this study.

**Plasmid**	**Description**	**Reference**
pMK1*lux*	pBR322 ori with the *luxCDABE* operon and MCS; Amp^R	Karavolos *et al*. 2008
pMK1*lux*-P_{LEE1Cr}	pMK1*lux* with the ICC168 LEE1 promoter cloned into the MCS; Amp^R	This study
pMK1*lux*-P_{aau}	pMK1*lux* with the ZAP193 *aau* promoter cloned into MCS; Amp^R	Cottam *et al*. 2024
pMK1*lux*-P_{24811}	pMK1*lux* with the ICC168 *rbl* promoter cloned into the MCS; Amp^R	This study
pSUPROM	Cloning vector for expression under the TatA promoter; Kan^R	Jack *et al*. 2004
pSU-*rbl*	pSUPROM with the full *rbl* locus cloned into MCS; Kan^R	This study
pSU-*aau*	pSUPROM with ZAP193 *aau* cloned into MCS; Kan^R	Cottam *et al*. 2024
pET28a	Expression vector for recombinant His-tagging; Kan^R	Lab stock
pET28a-*aauA*	pET28a with *aauA* (ZAP193 locus tag 0432; excluding signal peptide) cloned into the MCS; Kan^R	This study
pET28a-*araB*	pET28a with *araB* (from ZAP193) cloned into the MCS; Kan^R	This study
pKD46	Lambda Red recombinase expressing plasmid; temperature sensitive; Amp^R	Datsenko and Wanner, 2000
pKD3	Template plasmid for Lambda Red mutagenesis; Cm^R	Datsenko and Wanner, 2000
pKD4	Template plasmid for Lambda Red mutagenesis; Kan^R	Datsenko and Wanner, 2000
pCP20	FLP recombinase expressing plasmid; temperature sensitive; Amp^R	Datsenko and Wanner, 2000

Supplementary Table 4 – X-ray data collection and refinement statistics.

	**AauA dataset*<br>(PDBID: 9I1M)**
**Data collection:**	
Beamline	Diamond Light Source I03
Space group	C2
Cell dimensions	
a, *b*, *c* (Å)	76.38, 66.94, 57.85
α, β, γ (°)	90, 94.45, 90
Resolution (Å)	38.9-1.35 (1.37-1.35)
R_{pim}	0.018 (0.098)
R_{meas}	0.033 (0.14)
$I / \sigma I$	29.8 (7.5)
$CC_{1/2}$	0.999 (0.98)
Completeness (%)	79.1 (18.9)
Redundancy	6.2 (3.2)
**Refinement:**	
Resolution (Å)	1.35
No. reflections	50386 (574)
R_{work} / R_{free}	0.126/0.143
No. atoms	5509
Protein	4464
Ligand/ion	74
B-factors (Å^2)	
Protein	12.7
Ligand/ion	23.26
R.m.s. deviations	
Bond lengths (Å)	0.0124
Bond angles (°)	2.022
Rotamer outliers (%)	0.42
Ramachandran (%)<br>Favoured regions<br>Allowed regions<br>Outliers	 97.22<br>2.78<br>0
Molprobity score	1.66

*Values in parentheses are for highest-resolution shell.

Supplementary references:

Campellone, K.G., Giese, A., Tipper, D.J., and Leong, J.M. A tyrosine- phosphorylated 12-amino-acid sequence of enteropathogenic *Escherichia coli* Tir binds the host adaptor protein Nck and is required for Nck localization to actin pedestals. *Molecular Microbiology* 43*:*1227–1241 (2002).

Cottam C, White RT, Beck LC, Stewart CJ, Beatson SA, Lowe EC, Grinter R and Connolly JPR. Metabolism of L-arabinose converges with virulence regulation to promote enteric pathogen fitness. *Nature Communications* 15(1):4462 (2024).

Datsenko, K. A. and Wanner, B. L. One-step inactivation of chromosomal genes in *Escherichia coli* K-12 using PCR products. *Proceedings of the National Academy of Sciences U. S. A.* 97:6640–6645 (2000).

Jack, R. L. *et al.* Coordinating assembly and export of complex bacterial proteins. *EMBO Journal* 23, 3962–3972 (2004).

Karavolos, M. H. *et al.* Adrenaline modulates the global transcriptional profile of *Salmonella* revealing a role in the antimicrobial peptide and oxidative stress resistance responses. *BMC Genomics* 6:458 (2008).

Petty, N. K. *et al*. The *Citrobacter rodentium* genome sequence reveals convergent evolution with human pathogenic *Escherichia coli*. *Journal of Bacteriology* 192:525–538 (2010).

Roe, A. J. *et al.* Co-ordinate single-cell expression of *LEE4*- and *LEE5*-encoded proteins of *Escherichia coli* O157:H7. *Molecular Microbiology* 54(2):337-352 (2004).
