# Supplementary material for: Convergent evolution of distinct D-ribulose utilisation pathways in attaching and effacing pathogens
Source: Nat Commun. 2025 Jul 29;16:6976. doi: 10.1038/s41467-025-62476-5 (PMC12307932; doi:10.1038/s41467-025-62476-5)
Supplement: Supplementary file 2 — Reporting Summary [file 41467_2025_62476_MOESM2_ESM.pdf]

nature portfolio

Corresponding author(s): James Connolly

Last updated by author(s): Jul 15, 2025

Reporting Summary

Nature Portfolio wishes to improve the reproducibility of the work that we publish. This form provides structure for consistency and transparency in reporting. For further information on Nature Portfolio policies, see our Editorial Policies and the Editorial Policy Checklist.

Statistics

For all statistical analyses, confirm that the following items are present in the figure legend, table legend, main text, or Methods section.

n/a	Confirmed	
☐	☒	The exact sample size (*n*) for each experimental group/condition, given as a discrete number and unit of measurement
☐	☒	A statement on whether measurements were taken from distinct samples or whether the same sample was measured repeatedly
☐	☒	The statistical test(s) used AND whether they are one- or two-sided<br>*Only common tests should be described solely by name; describe more complex techniques in the Methods section.*
☐	☒	A description of all covariates tested
☐	☒	A description of any assumptions or corrections, such as tests of normality and adjustment for multiple comparisons
☐	☒	A full description of the statistical parameters including central tendency (e.g. means) or other basic estimates (e.g. regression coefficient) AND variation (e.g. standard deviation) or associated estimates of uncertainty (e.g. confidence intervals)
☐	☒	For null hypothesis testing, the test statistic (e.g. *F*, *t*, *r*) with confidence intervals, effect sizes, degrees of freedom and *P* value noted<br>*Give P values as exact values whenever suitable.*
☒	☐	For Bayesian analysis, information on the choice of priors and Markov chain Monte Carlo settings
☐	☒	For hierarchical and complex designs, identification of the appropriate level for tests and full reporting of outcomes
☒	☐	Estimates of effect sizes (e.g. Cohen's *d*, Pearson's *r*), indicating how they were calculated

Our web collection on statistics for biologists contains articles on many of the points above.

Software and code

Policy information about availability of computer code

Data collection	Please see methods section for details of data collection.
Data analysis	Please see methods section for full details of data analysis tools used along with accompanying citations. Packages include - GraphPad Prism v10; MEGA11 v11.0.12; PyMol v2.6; SignalP v5.0; AutoDock Vina v1.2; Phenix eLBOW v1.20.1; Coot 0.9.8.93; MolRep v11.0; Microcal PEAQ-ITC v1.4; Aimless v0.8.2; REFMAC v5.8.0425; AutoDock (MGL) tools v1.5.7

For manuscripts utilizing custom algorithms or software that are central to the research but not yet described in published literature, software must be made available to editors and reviewers. We strongly encourage code deposition in a community repository (e.g. GitHub). See the Nature Portfolio guidelines for submitting code & software for further information.

Data

Policy information about availability of data

All manuscripts must include a data availability statement. This statement should provide the following information, where applicable:

- Accession codes, unique identifiers, or web links for publicly available datasets
- A description of any restrictions on data availability
- For clinical datasets or third party data, please ensure that the statement adheres to our policy

The X-ray crystal structure of AauA has been deposited to the Protein Data Bank under the accession ID 9I1M (https://doi.org/10.2210/pdb9I1M/pdb). Source data for Figs. 1-6, Supplementary Figs. 1-6 and 10-12 are provided with this paper as a Source Data file. Publicly available genome sequences used in Fig. 6 and Supplementary Fig. 11 were obtained from the NCBI Sequence Read Archive and the Integrated Microbial Genomes and Microbiomes database respectively with

the corresponding accession numbers provided in the Source Data file.

Research involving human participants, their data, or biological material

Policy information about studies with human participants or human data. See also policy information about sex, gender (identity/presentation), and sexual orientation and race, ethnicity and racism.

Reporting on sex and gender	N/A
Reporting on race, ethnicity, or other socially relevant groupings	N/A
Population characteristics	N/A
Recruitment	N/A
Ethics oversight	N/A

Note that full information on the approval of the study protocol must also be provided in the manuscript.

Field-specific reporting

Please select the one below that is the best fit for your research. If you are not sure, read the appropriate sections before making your selection.

- [x] Life sciences
- [ ] Behavioural & social sciences
- [ ] Ecological, evolutionary & environmental sciences

For a reference copy of the document with all sections, see nature.com/documents/nr-reporting-summary-flat.pdf

Life sciences study design

All studies must disclose on these points even when the disclosure is negative.

Sample size	Sample sizes were determined based on our own published data and the extensive literature utilizing the methods described, in order to support the appropriate statistical analyses performed.
Data exclusions	We did not need to exclude any data.
Replication	All in vitro experiments were performed in biological triplicate on separate occasions (with technical replicates where appropriate described in the methods) unless otherwise specified. All attempts to replicate results were successful.
Randomization	N/A; samples were not allocated to experimental groups
Blinding	N/A; samples were not allocated to experimental groups

Reporting for specific materials, systems and methods

We require information from authors about some types of materials, experimental systems and methods used in many studies. Here, indicate whether each material, system or method listed is relevant to your study. If you are not sure if a list item applies to your research, read the appropriate section before selecting a response.

Materials & experimental systems

n/a	Involved in the study	
[ ]	[x]	Antibodies
[x]	[ ]	Eukaryotic cell lines
[x]	[ ]	Palaeontology and archaeology
[x]	[ ]	Animals and other organisms
[x]	[ ]	Clinical data
[x]	[ ]	Dual use research of concern
[x]	[ ]	Plants

Methods

n/a	Involved in the study	
[x]	[ ]	ChIP-seq
[x]	[ ]	Flow cytometry
[x]	[ ]	MRI-based neuroimaging

Antibodies

Antibodies used	Anti-His HRP-conjugated antibodies are monoclonal and were generated in mice. These are commercial antibodies and were purchased from antibodies.com (A1057). Antibodies were used at a concentration of 1:5000 for Western blot analysis.

Validation	Validation of these antibodies was performed using previously confirmed His-tagged recombinant proteins from our stocks as well as untagged control strains.

Plants

Seed stocks	N/A
Novel plant genotypes	N/A
Authentication	N/A
